# The effectiveness of a social media intervention for reducing portion sizes in young adults and adolescents

**DOI:** 10.1177/2055207619878076

**Published:** 2019-09-22

**Authors:** Maxine A Sharps, Marion M Hetherington, Pam Blundell-Birtill, Barbara J Rolls, Charlotte EL Evans

**Affiliations:** 1 School of Applied Social Sciences, De Montfort University, UK; 2 School of Psychology, University of Leeds, UK; 3 Department of Nutritional Sciences, The Pennsylvania State University, USA; 4 School of Food Science and Nutrition, University of Leeds, UK

**Keywords:** Social norms, peers, eating behaviour, nutrition, nudging

## Abstract

**Objective:**

Adolescents and young adults select larger portions of energy-dense food than recommended. The majority of young people have a social media profile, and peer influence on social media may moderate the size of portions selected.

**Methods:**

Two pilot interventions examined whether exposure to images of peers’ portions of high-energy-dense (HED) snacks and sugar-sweetened beverages (SSBs) on social media (Instagram) would influence reported desired portions selected on a survey. Confederate peers posted ‘their’ portions of HED snacks and SSBs on Instagram. At baseline and intervention end participants completed surveys that assessed desired portion sizes.

**Results:**

In intervention 1, undergraduate students (*n* = 20, mean age=19.0 years, SD=0.65) participated in a two-week intervention in a within-subjects design. Participants reported smaller desired portions of HED snacks and SSBs following the intervention, and smaller desired portions of HED snacks for their peers. In intervention 2, adolescents (*n* = 44, mean age = 14.4 years, SD = 1.06) participated in a four-week intervention (*n* = 23) or control condition (*n* = 21) in a between-subjects design. Intervention 2 did not influence adolescents to reduce their reported desired portion sizes of HED snacks or SSBs relative to control.

**Conclusions:**

These preliminary studies demonstrated that social media is a feasible way to communicate with young people. However, while the intervention influenced young adults’ reported desired portions and social norms regarding their peers’ portions, no significant impact on desired reported portion sizes was found for HED snacks and SSBs in adolescents. Desired portion sizes of some foods and beverages may be resistant to change via a social media intervention in this age group.

## Introduction

Food and beverage portion sizes have increased in recent years^1,2^ and there is robust evidence that adults and children eat more when served a larger portion than when served a smaller portion.^3–7^ In particular, high-energy-dense (HED) foods such as sweet and savoury snacks, and sugar-sweetened beverages (SSBs) have been shown to be chosen in larger portions than recommended,^8,9^ with adolescents preferentially selecting these items.^9^ Hollands et al. suggest that reduced exposure to larger than recommended portions across the diet could reduce energy intake by 12–16% in adults and children.^4^ Therefore, finding strategies to reduce exposure and to encourage selection of smaller portions of HED snacks and SSBs is an important next step.^7^

Social media is widely used, with 2.89 billion active users as of 2017^10^ and 74% of adolescents having a social media profile.^11^ A recent study found that the majority of images (67.7%) posted by adolescents on social media were of HED snack foods.^12^ Therefore, social media may be a valuable intervention tool for encouraging the selection of smaller portions of HED snacks and SSBs. There is evidence that incorporating peers in a social media intervention may improve young adults’ sexual health knowledge and behaviour;^13,14^ however, less is known about the influence of peers on social media for eating behaviour.

According to the normative model of social influence^15^ people are often uncertain about how to act in a situation, and rely on the behaviour of others for guidance when such behaviours are salient. Peers are known to be a key influence on eating behaviour in experimental studies,^16–20^ and people have been shown to adjust their eating behaviour to that of a present instructed confederate peer,^21–23^ to remote peers who are visible but not present,^18^ and to social norms which indicate the behaviour of others.^24^ For example, a peer on a video influenced adolescents’ food intake, with adolescents eating more when the video peer ate a large amount, and less when the video peer ate a small amount.^18^ Furthermore, exposing participants to information about how other people in the study have eaten (e.g. an information sheet which states the amount of food eaten by other people) has been shown to influence eating behaviour.^24^ Thus, it is plausible that images of remote confederate peers’ snacks and drinks on social media may set a social norm and influence other people’s portion sizes. However, to our knowledge this has not been examined and warrants investigation.

Here, two pilot interventions examined the feasibility of a social media intervention which involved exposure to images of peers’ portions of HED snacks and SSBs (which depicted the recommended portion size), as a way of reducing participants’ own self-reported desired portion sizes of HED snacks and SSBs. The influence of the intervention on participants’ perceptions of their peers’ portions (social norms) was also examined. Pilot intervention 1 assessed the feasibility of this intervention in young adults and pilot intervention 2 in adolescents. Based on the normative model of social influence^15^ and previous social norm studies,^17,18,25,26^ it was hypothesised that viewing images of peers’ portions of HED snacks and SSBs (which depicted the recommended portion) via social media would reduce self-reported desired portion sizes of HED snacks and SSBs.

## Methods

### Participants in pilot intervention 1

Undergraduate Psychology students (*n* = 21) were recruited from the University of Leeds Psychology research participation system and received study credit for taking part. The study was advertised on the research participation system for one week in March 2017^a^ until a sufficient number of participants were recruited. A power calculation was not conducted in either intervention since these were pilot interventions designed to test feasibility. In intervention 1 we aimed to recruit a minimum of 20 participants. One participant was excluded due to not completing the second survey. The final sample consisted of 20 young adults (19 females, 1 male) aged 18–20 years (mean = 19.00, SD = .65). One participant did not enter their height and weight and so their body mass index (BMI) could not be calculated. Of the 19 participants whose BMI was calculated, the majority were classed as having a BMI within the healthy range (70% healthy weight, mean = 22.17, SD = 2.54).

### Participants in pilot intervention 2

**Figure 1.**
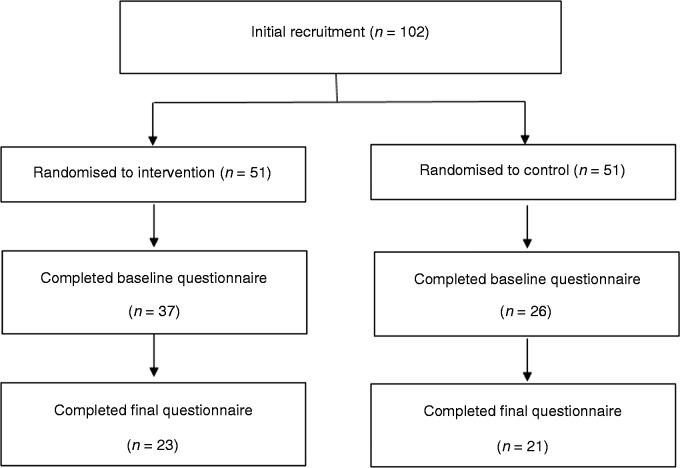
Intervention 2 participant recruitment and retention flowchart.

The intervention was advertised to 16-year-olds and parents of 13–16-year-old adolescents on social media (Facebook)^b^ over a three-week period in April 2017 until a sufficient number of participants had been recruited. Those interested in the research were asked to contact the researcher via email or on Facebook. Parents were provided with an information sheet which fully informed them of the study aims and procedures. Parents assented to their adolescent child participating through providing their adolescent child with the details of the research if they were happy for them to take part. All adolescents who were interested in the research emailed the researcher and were provided with a link to the baseline survey where they were required to read an information sheet and provide their consent. Due to potential dropout we aimed to recruit a minimum of 100 adolescents (50 per condition). A total of 102 adolescents were recruited from Facebook and the final sample consisted of 44 adolescents (23 intervention, 21 control; 31 females, 13 males), aged 13–16 years (mean = 14.36, SD = 1.06) (see Figure 1 for the participant recruitment and retention flowchart). Ten adolescents did not self-report their height and weight. Of the 34 who did, the majority were classed as having a BMI within the healthy range (85.3% healthy weight, mean BMI = 20.63, SD = 3.85). Adolescents received a £10 voucher for participating in the intervention.

### Design of interventions 1 and 2

Intervention 1 lasted for two weeks and used a 2 × 2 within-subjects repeated-measures design, with factors food type (HED snacks and SSBs) and time (baseline and intervention end). Intervention 2 lasted for four weeks and employed a 2 × 2 × 2 mixed design, with a between-subjects factor of condition (intervention versus control) and within-subjects factors of food type (HED snacks and SSBs) and time (baseline and intervention end). In intervention 2 adolescents were randomly allocated to a condition (the lead author randomised participant numbers to a condition (using randomizer.org) and adolescents were allocated to a condition based on the order in which they contacted the lead author). In both interventions all participants were informed that the intervention was examining snacking behaviour but were not informed that the research was investigating portion sizes. Surveys were completed at baseline and at the end of the intervention to examine whether the intervention reduced desired portion size. The survey also examined whether the intervention influenced participants’ perceptions of their peers’ ‘desired’ portion sizes, as well as participants’ frequency of consumption, liking, and intentions regarding their portions of HED snacks and SSBs.

**Figure 2.**
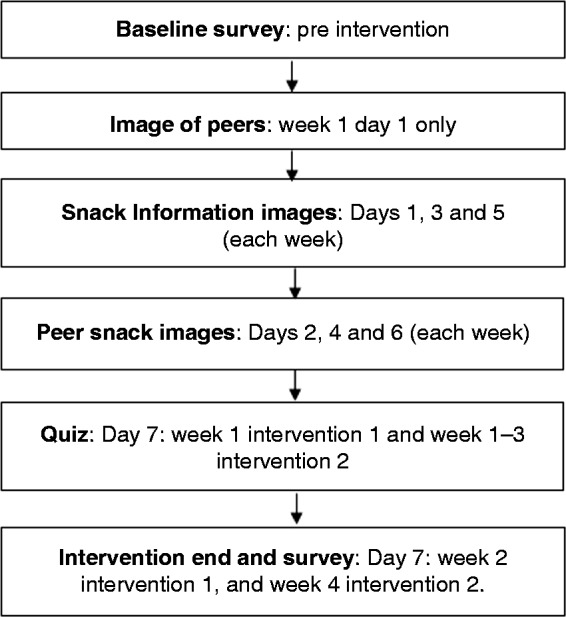
Intervention content posting timeline for interventions 1 and 2.

**Figure 3.**
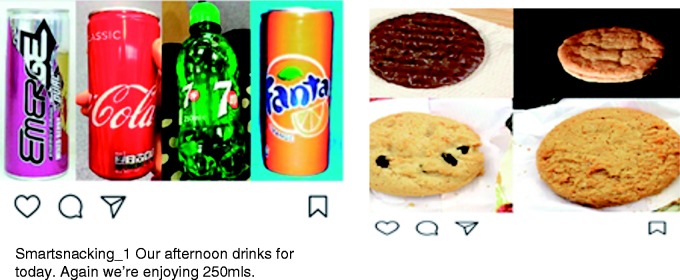
Peer high-energy-dense snack and sugar-sweetened beverage images for interventions 1 and 2.

In the intervention conditions (all participants in intervention 1, and only intervention condition participants in intervention 2) one confederate peer (who was a member of the research team) posted daily on the behalf of all four confederate peers in a joint Instagram account called Smart Snacking. The images of the same four confederate peers (two females and two males) were used in both interventions. The images showed the peers when they were 18–20 years old in intervention 1 and 16–18 years old in intervention 2. We opted to show the peers within these age ranges as research has shown that people model on peers of a similar age or older than themselves.^27^ (This was achieved by the confederate peers providing images of themselves between the ages of 16–18 years and 18–20 years.^c^) Participants were not aware that the peers were confederates. Each week the confederate peer posted images of the four peers’ portions of HED snacks or SSBs (which constituted the recommended portion).^d^ The confederate peer also posted images of content related to snacking and portion size such as snack information images (including calorie information, sugar content and portion size information of popular snacks) and quizzes (see Figure 2 for the intervention posting timeline). The snack information images and the quizzes were only included to corroborate the cover story that the intervention was looking at snacking behaviour. All peer portion images were created by the experimenter and were not the peers’ actual snack or SSB images. The peer portion images contained the snack/SSB for all four peers and were presented with the pronoun ‘our’ and were not linked to a particular peer (see Figure 3).^e^ Week 1 of both interventions focussed on cookies/biscuits, week 2 on SSBs, and week 3 and 4 of intervention 2 focussed only on savoury snacks and confectionery respectively. Participants in the control condition only completed the baseline surveys and were emailed the quizzes.

### Procedure

Interested participants were emailed a link to access the survey hosted on Bristol Online Surveys (https://www.onlinesurveys.ac.uk). Participants were given information and invited to consent to participation. Participants in the intervention conditions were asked to enter their Instagram username at the end of the baseline survey. Once the required number of participants were recruited, participants in the intervention conditions were added to the Instagram account and the intervention began. Participants in the intervention conditions were required to log on daily and to like every post, and all participants (intervention and control) were required to complete the weekly quizzes. A link was provided to the quizzes in the Instagram group for the intervention participants and was emailed to the control condition participants. At the end of the intervention participants completed the end of intervention survey. Upon completion of the study a debrief statement and study credit (intervention 1)/payment (intervention 2) were sent to participants.

### Survey

#### Participants’ desired portion sizes and perceptions of their peers’ desired portion sizes

To set the scene for the survey, participants were told to ‘Imagine it is 3pm in the afternoon. You had a sandwich for your lunch at 12 noon, and you still have a few hours before the evening meal and you are about to have a snack’. For SSBs, participants were presented with the statement ‘Imagine that it is 5pm in the afternoon and you decide to have a drink’. For each image, judgements were made on whether the portion was ‘too little’, ‘slightly less than I would eat’, ‘just right’, ‘slightly more than I would eat’ or ‘too much’. See supplementary material for information about the snacks and SSBs and how desired portion sizes were calculated; see Table 1 for energy and macronutrient content of the HED snacks and SSBs.

**Table 1.** Energy content and macronutrient content of high-energy-dense snacks and sugar-sweetened beverages used in the intervention pictures.

	Recommended portion^a^	Energy/macronutrient content per portion and per 100g											
		Energy kcal (kJ)		Fat^b^ (saturated fat)		Carbohydrate^b^ (of which sugars)		Fibre^b^		Protein^b^		Salt^b^	
Food item		Per portion	Per 100g	Per portion	Per 100g	Per portion	Per 100g	Per portion	Per 100g	Per portion	Per 100g	Per portion	Per 100g
HED snacks													
Chocolate buttons	25g	134 (558.5)	535.0 (2234.0)	7.5 (4.5)	30.0 (18)	14.25 (14)	57.0 (56.0)	0.5	2.1	1.8	7.3	0.05	0.2
Chocolate digestive	16.7g	83 (346)	495.0 (2071.0)	3.9 (2.1)	23.6 (12.4)	10.4 (4.9)	62.2 (29.5)	0.5	3.0	1.1	6.7	0.2	1.0
Jelly sweets	29g	97 (414)	334.0 (1420.0)	Trace	0.1 (0.1)	22.6 (15.5)	77.4 (53.1)	0.3	1.1	1.6	5.4	0.01	0.03
Chocolate chip cookies	21g	104 (438)	491.0 (2059.0)	4.7 (2.4)	22.1 (11.3)	13.9 (7.3)	65.4 (34.4)	0.7	3.1	1.2	5.8	0.12	0.6
Mini chocolate chip muffins	25g	109 (456)	436.0 (1823.0)	5.6 (0.9)	22.5 (3.6)	13.1 (7.1)	52.5 (28.4)	<0.5	1.6	1.3	5.0	0.09	0.3
Swiss roll	32g	113 (477)	353 (1492)	2.5 (1.7)	7.8 (5.3)	21.3 (14.1)	66.6 (44.1)	0.4	1.4	1.1	3.5	0.2	0.7
Chocolate cake	87.5g	286 (1196)	433.0 (1812)	14.0 (3.8)	21.0 (5.7)	36.0 (21.0)	55.0 (32.0)	1.4	2.1	2.8	4.3	0.2	0.3
Salted popcorn	25g	135 (562)	537.0 (2240.0)	7.4 (0.6)	29.4 (2.3)	13.7 (0.3)	54.6 (1.2)	2.4	9.6	2.1	8.5	0.3	1.2
Pretzels	30g	118 (499)	393.0 (1662.0)	1.4 (0.2)	4.6 (0.5)	23 (1.0)	76.0 (3.3)	1.1	3.6	3.0	10.0	0.75	2.5
Ready salted crisps	25g	132 (548)	526.0 (2194.0)	8.0 (0.7)	31.9 (2.6)	12.9 (0.1)	51.5 (0.4)	1.1	4.3	1.5	6.1	0.4	1.4
SSBs													
Full sugar cola	250ml	105 (105)	42.0 (180.0)	0.0 (0.0)	0.0 (0.0)	27.0 (27.0)	10.6 (10.6)	0	0	0	0	0	0
Full sugar cordial drink	250ml	52 (223)	21.0 (89.0)	0.0 (0.0)	0.0 (0.0)	11.9 (11.6)	4.8 (4.6)	0	0	0	0	0.14	0.06
Energy drink	250ml	115 (485)	46.0 (194.0)	0.0 (0.0)	0.0 (0.0)	27.5 (27.5)	11.0 (11.0)	0	0	0	0	0.25	0.1
Chocolate milkshake	250ml	187.5 (792.5)	75.0 (317.0)	3.75 (2.75)	1.5 (1.1)	27.5 (27.5)	11.0 (11.0)	<0.5	<0.5	9.75	3.9	0.25	0.1

^a^The recommended portion is based on the manufacturers’ recommendations.

^b^Fat, carbohydrate, fibre, protein and salt content are reported in grams.

HED: high-energy-dense; SSB: sugar-sweetened beverage.

#### Frequency of consumption, liking and intentions

Participants’ reported frequency of consumption and liking of each item and intentions were assessed based on questions used by Stok et al.^28^ (see supplementary material). Mean frequency, liking and intention scores were calculated for HED snacks and SSBs at baseline and intervention end. A low score for frequency indicated that the item was not eaten frequently, a low score for liking indicated that the item was not liked and a low intention score indicated that participants did not intend to change their behaviour.

### Ethics

Interventions 1 and 2 received ethical approval from the School of Psychology University of Leeds Research Ethics Committee, Faculty of Medicine and Health (ref: 17-0094 and 17-0001).

### Statistical analysis

#### Main analysis

In intervention 1 the main planned analysis was a 2 (food type: HED snacks and SSBs) × 2 (time: baseline and intervention end) repeated-measures analysis of variance (ANOVA). In intervention 2 the main planned analysis was a 2 × 2 × 2 mixed ANOVA with a between-subjects factor of condition (intervention versus control), and within-subjects factors of food type (HED snacks and SBBs) and time (baseline and intervention end). In both interventions the dependent variables were participants’ self-reported ‘desired’ portion sizes of HED snacks and SSBs. We planned to examine the main effects of the independent variables and any interactions between these. Across both interventions we made an a priori decision to control for age and standardised zBMI; however, due to the small sample sizes, and since these variables did not correlate with the dependent variables, we opted not to control for these variables in the main or additional analysis. Gender did not correlate with the dependent variables (*p* > .05) and was not controlled for in any of the analyses, and removing the one male from the analysis in intervention 1 did not alter the results, therefore the results reported include the male. (See supplementary material for the analysis adjusted by age and standardised BMI, and with the male participant removed).

#### Additional analysis

Separate ANOVAs (2 × 2 repeated-measures ANOVAs in intervention 1 and 2 × 2 × 2 mixed ANOVAs in intervention 2) were conducted to examine the influence of the intervention on participants’ perceptions of their peers’ desired portion sizes of HED snacks and SSBs, and participants’ frequency of consumption, liking, and intentions regarding their portions of HED snacks and SSBs.

HED snack and SSB items which were rated as less than 3 for liking were not included in the analysis for participants’ desired portion sizes, frequency of consumption and liking. In intervention 1 energy drinks (mean = 2.29, SD = 1.35) were excluded from the analysis. In intervention 2 energy drinks (mean = 2.29, SD = 1.28), pretzels (mean = 1.27, SD = .77), and jelly sweets (mean = 2.24, SD = 1.29) were excluded from the analysis. See Table 2 for means and SDs for results of intervention 1 and Table 3 for means and SDs for results of intervention 2.

**Table 2.** Participants’ mean (SD) desired portion sizes, perceptions of peers’ desired portion sizes, frequency of consumption, liking and intentions regarding participants’ high-energy-dense snack and sugar-sweetened beverage intake for intervention 1.

	HED snacks		SSBs	
	Baseline	Intervention end	Baseline	Intervention end
Participants’ desired portion size^a^	1.47 (.28)^b^	1.28 (.27)^b^	.88 (.21)^b^	.81 (.27)^b^
Perceptions of peers’ desired portion size^a^	1.46 (.26)^b^	1.34 (.28)^b^	.85 (.23)	.89 (.25)
Frequency of consumption^c^	1.58 (.33)	1.51 (.45)	2.12 (.78)	1.98 (.81)
Liking^c^	3.97 (.40)	3.93 (.33)	3.77 (.63)	3.87 (.46)
Intentions^d^	3.53 (1.03)	3.88 (.92)	2.80 (1.02)	3.18 (.98)

^a^For desired portion size, a value of 1 refers to the recommended portion size for HED snacks and the typical portion for SSBs. A number greater than 1 indicates the ‘desired’ portion size is greater than the recommended portion, and a number smaller than 1 indicates that the ‘desired’ portion size is smaller than the recommended portion.

^b^Indicates a significant difference between baseline and intervention end.

^c^Frequency of consumption was measured on a 6-point Likert-style scale from once per month or never to daily. Liking was measured on a 5-point Likert scale from strongly dislike to strongly like.

^d^Intentions were assessed on a 5-point Likert-style scale from completely disagree to completely agree.

HED: high-energy-dense; SSB: sugar-sweetened beverage.

**Table 3.** Mean (SD) participants’ reports of desired portion sizes, perceptions of peers’ desired portion sizes, frequency of consumption, liking and intentions regarding participants’ high-energy-dense snack and sugar-sweetened beverage intake for intervention 2.

	HED snacks						SSBs	
	Intervention		Control		Intervention		Control	
	Baseline	Intervention end	Baseline	Intervention end	Baseline	Intervention end	Baseline	Intervention end
Participants’ desired portion size^a^	1.28 (.34)	1.25 (.35)	1.36 (.31)	1.38 (.33)	.86 (.27)	.86 (.28)	.93 (.33)	.87 (.34)
Perceptions of peers’ desired portion size^a^	1.40 (.36)	1.38 (.36)	1.44 (.33)	1.49 (.27)	.93 (.25)	.96 (.24)	.98 (.27)	.93 (.31)
Frequency of consumption^b^	2.05 (.51)	2.13 (.73)	2.01 (.55)	1.92 (.47)	2.28 (.81)	2.29 (.81)	2.18 (.93)	1.95 (.93)
Liking^b^	4.08 (.52)	4.05 (.52)	4.07 (.52)	3.84 (.87)	3.91 (.78)	3.72 (.89)	3.77 (1.03)	3.48 (1.15)
Intentions^c^	3.53 (.96)	3.33 (.98)	3.19 (.84)	3.13 (.76)	3.26 (1.10)	3.17 (.95)	3.08 (.88)	2.95 (.79)

^a^For desired portion size, a value of 1 refers to the recommended portion size of HED snacks and the typical portion size of SSBs. A number greater than 1 indicates the ‘desired’ portion size is greater than the recommended/typical portion, and a number smaller than 1 indicates that the ‘desired’ portion size is smaller than the recommended/typical portion.

^b^Frequency of consumption was measured on a 6-point Likert-style scale from once per month or never to daily. Liking was measured on a 5-point Likert scale from strongly dislike to strongly like.

^c^Intentions were assessed on a 5-point Likert-style scale from completely disagree to completely agree.

HED: high-energy-dense; SSB: sugar-sweetened beverage.

## Results

### Main analysis of intervention 1

#### Participants’ reported desired portion sizes

There was a significant main effect of time (F (1, 19) = 14.68, *p* = .001, ƞp^2^ = .44). Participants reported smaller desired portion sizes of HED snacks and SSBs at intervention end than at baseline. There was no significant food type by time interaction (F (1, 19) = 3.70, *p* = .07, ƞp^2^ = .16) on participants’ desired portion sizes of HED snacks and SSBs between baseline and intervention end. The results indicate that exposure to the intervention influenced participants to reduce their self-reported desired portion sizes of HED snacks and SSBs following the intervention.

### Additional analysis of intervention 1

#### Reported perceptions of their peers’ desired portion sizes

A significant main effect of food type (F (1, 19) = 64.72, *p* = .001, ƞp^2^ = .77) but no significant main effect of time (F (1, 19) = 1.56, *p* = .23, ƞp^2^ = .08) were found. A significant food type × time interaction (F (1, 19) = 4.68, *p* = .04, ƞp^2^ = .20) on participants’ perceptions of their peers’ portion sizes of HED snacks and SSBs was found. Paired samples *t*-tests indicated that participants reported smaller HED portion sizes for their peers at intervention end than at baseline, *t* (19) = 2.26, *p* = .04, but not for SSBs.

#### Reported frequency of consumption and liking and intentions

For frequency of consumption, there was a significant main effect of food type (F (1, 19) = 9.57, *p* = .006, ƞp^2^ = .34). Participants reported consuming SSBs more frequently than HED snacks. There were no other significant main effects or interactions (*p* > .05) on participants’ frequency of consumption, liking or intentions regarding their HED snacks or SSBs between baseline and intervention end.

### Main analysis of intervention 2

#### Participants’ reported desired portion sizes

There was no significant main effect of condition (F (1, 41) = .92, *p* = .34, ƞp^2^ = .02), no significant main effect of time (F (1, 41) = .58, *p* = .45, ƞp^2^ = .01) and no significant interactions (*p* > .05). Thus, the intervention did not influence participants to reduce their desired portion sizes of HED snacks or SSBs relative to the control condition.

### Additional analysis of intervention 2

#### Reported perceptions of their peers’ desired portion sizes

There was no significant main effect of condition (F (1, 41) = .43, *p* = .52, ƞp^2^ = .01), and no other significant main effects or interactions (*p* >.05*)* on participants’ perceptions of their peers’ portion sizes of HED snacks and SSBs between baseline and intervention end. The intervention did not significantly influence participants’ perceptions of their peers’ desired portion sizes of HED snacks or SSBs relative to the control condition.

#### Reported frequency of consumption and liking and intentions

There were no significant main effects or interactions (*p* > .05) for frequency of consumption, liking or intentions.

## Discussion

In this paper we piloted a novel social media intervention which aimed to reduce participants’ self-reported desired portion sizes of HED snacks and SSBs using peer influence. Intervention 1 showed a significant reduction in young adults’ reported desired portions of HED snacks and SSBs following the intervention. Intervention 1 also influenced young adults’ social norms, whereby there was a significant reduction in participants’ perceptions of their peers’ HED snack portions following the intervention. However, intervention 2 did not significantly influence adolescents’ reported desired portions, or their perceptions of their peers’ desired portions of HED snacks and SSBs. Although these interventions are pilots and further research is needed, the results indicate that a social media intervention using peer influence may be a potential strategy for shifting social norms and downsizing self-reported desired portions in young adults.

Intervention 2 may not have influenced adolescents’ desired portion sizes due to the type of peer used as an influencer. According to the normative model of social influence, people look to others for guidance on how to behave in situations which they are unfamiliar with; however, only when such examples are salient.^15^ No information was given about the peers in the interventions, which is consistent with previous research,^18^ and appeared to be sufficient for young adults. The intervention did not influence adolescents’ perceptions of their peers’ desired portions, suggesting that the peers may not have been salient for the adolescents. Research has shown that popular peers were perceived to eat more healthily than unpopular peers,^29,30^ and the more that the participants identified with their popular peers, the more healthily they ate.^30^ Since middle adolescents (aged 13–17 years) have been shown to be the least susceptible to peer influence,^31^ the peers used in such interventions may need to be particularly salient in order to influence middle adolescents’ behaviour. Thus, using popular peers that the adolescents identify with (e.g. popular peers at their school) may influence adolescents’ behaviour and would be a valuable avenue to pursue in a future intervention.

Social norms refer to codes of conduct about how to behave.^32^ Descriptive social norms describe the behaviour of others,^33^ and can be communicated through present and remote peers and have been shown to influence eating behaviour.^23,24,34^ However, people often misperceive descriptive social norms and these misperceptions can impact behaviour.^35,36^ For example, adolescents (16–19-year-olds) have been shown to overestimate peers’ intake of HED snacks by 1.8 portions, and SSBs by 5.2 portions per week, and these overestimations were strongly associated with the adolescents’ own intake of SSBs and HED snacks.^35^ Therefore, correcting social norm misperceptions is important, and targeting social norm misperceptions may be a valuable first step to changing behaviour. Intervention 1 showed that descriptive social norms provided by remote peers on social media positively shaped young adults’ social norms regarding their peers’ portion sizes, with young adults reducing their perceptions of their peers’ desired portions at the end of the intervention. Therefore, this type of intervention may be a way of correcting normative misperceptions regarding peers’ portions in young adults. Furthermore, since social media is widely used,^10^ this type of intervention may have the potential to correct misperceptions on a large scale. However, further research is required to examine the impact of this type of intervention on normative misperceptions in a larger sample and over a longer period of time.

Considering that 74% of 12–15-year-olds have a social media profile,^11^ and there were 2.89 billion active social media profiles as of June 2017,^10^ finding ways to utilise social media in research into eating behaviour is important. Intervention 2 supports the use of social media as a recruitment tool for adolescents, as 102 adolescents were recruited through advertising to 16-year-olds and parents of 13–16-year-olds on social media. However, only 43% of the adolescents completed the intervention, indicating that retaining adolescents in interventions is a challenge and over-recruitment may be necessary to help to maintain participant numbers throughout the intervention. One challenge of social media–based interventions is the reliance on self-report. It has been shown that participants can estimate portion sizes from photographic images;^37,38^ however, participants were asked to identify a ‘desired’ portion size in these interventions, which may be open to a wider interpretation than estimating a weight. Using a validated dietary assessment tool specifically designed for assessing intake of energy-dense foods and developing a standardised system for assessing the effectiveness of social media on behaviour such as eating would be valuable in future research. Although a large number of people use social media,^10^ research has shown that certain people are more likely to use social media than others,^39^ which may result in a biased sample. For example, while males and females were equally likely to use social media, certain personality traits such as extraversion and openness to experience were linked to social media use.^39^ Therefore, understanding bias associated with social media samples is important.

In these interventions the adverts stated that we were examining snacking behaviour, which may attract a certain type of person, and may explain why the majority of participants had a healthy weight in both interventions. There was also only one male in intervention 1, which may also be related to the subject matter. Therefore, it is unclear whether young adult males and people who would benefit the most from the intervention (e.g. those with overweight and obesity), would be motivated to participate in a study investigating snacking. An examination of this approach with participants with overweight or obesity and with young adult males would be of value. Another consideration is that although these interventions focussed on peer influence, there were also components such as nutrition information and quizzes. Since intervention 1 did not include a control group, and intervention 2’s control group only completed quizzes and surveys, it is not possible to tease apart the effect of the nutrition information from the peer snack images, and to understand whether viewing images of snacks and drinks may have elicited priming effects. Therefore, in future research, including a control group where participants receive nutrition information and images without a reference to peers would allow for the examination of peer influence over and above the other intervention components. Furthermore, since the control group only completed quizzes and surveys, the amount of contact time of the intervention differed between the intervention and control group. Including a control group who are exposed to an Instagram account showing images unrelated to food would be of value in future studies. Finally, both interventions had small sample sizes; therefore we may have been underpowered to detect significant interactions. Investigating this approach with larger sample sizes in both interventions would be beneficial.

In conclusion, a social media intervention which involved briefly exposing young adults to images of confederate peers’ portion sizes of HED snacks and SSBs influenced a reduction in self-reported desired portion sizes of HED snacks and SSBs. Furthermore, the intervention also influenced young adults’ social norms regarding their peers’ desired portions, with participants indicating smaller desired portions of HED snacks for their peers at intervention end than baseline. This intervention did not influence adolescents’ self-reported desired portions. Future investigations with different types of peers and in populations with overweight and obesity would be of value to further evaluate the potential effects of a social media intervention utilising peer influence on adolescents’ and young adults’ eating behaviour.

## Supplemental Material

DHJ878076 Supplemental Material - Supplemental material for The effectiveness of a social media intervention for reducing portion sizes in young adults and adolescents Click here for additional data file. Supplemental material, DHJ878076 Supplemental Material for The effectiveness of a social media intervention for reducing portion sizes in young adults and adolescents by Maxine A Sharps, Marion M Hetherington, Pam Blundell-Birtill, Barbara J Rolls and Charlotte EL Evans in Digital Health

## Notes

a The advert stated that participants were required for a two-week snacking intervention and must be aged 18 years or over.

b The lead author joined multiple Facebook groups targeted at parents and advertised the study to parents of 13–16-year-old children and 16-year-olds within these groups and on the lead author’s personal Facebook profile. The adverts were not targeted at a specific geographic region or gender. The advert provided details about the intervention (i.e. a four-week snacking study) and that we were looking for 13–16-year-olds to participate and that they would receive a voucher for participating.

c The confederate peers were friends of the lead author who consented to their photographs being used for the purpose of the project.

d The HED snack images were always presented on a plate or napkin, while the SSBs were always presented as a can or bottle. The peers explicitly stated the portion size of the SSBs (250ml) to avoid any ambiguity about the portion size of the can/bottle. However, the peers did not state the portion size of the HED snacks as these were not deemed to be ambiguous.

e The peers were always shown to be eating the same type of snack (e.g. all the peers had a biscuit as their snack in week 1) because research has shown that ambiguous norms do not influence eating behaviour;^40^ therefore, we wanted the norm to be as clear as possible, and displaying a different type of snack for each peer may produce an ambiguous norm.
