# Supplementary material for: The effectiveness of a social media intervention for reducing portion sizes in young adults and adolescents
Source: Digit Health. 2019 Sep 22;5:2055207619878076. doi: 10.1177/2055207619878076 (PMC6757490; doi:10.1177/2055207619878076)
Supplement: DHJ878076 Supplemental Material - Supplemental material for The effectiveness of a social media intervention for reducing portion sizes in young adults and adolescents [file DHJ878076_Supplemental_Material.pdf]

Supplementary material

HED snack and SSB information

The HED foods and SSBs were selected because they are foods and drinks which are frequently overconsumed by this age range. The HED snacks consisted of sweet and savoury snacks and the SSBs consisted of soda, squash, energy drinks, and milkshake. For the snack foods, photographs were taken of four portions to represent half a portion, one portion, one and a half portions, and twice the recommended portion, which were weighed (in grams) and then plated for photography. Snacks were presented on a white 27cm (diameter) plate with a knife and fork on either side to provide size perspective. For the SSBs, photographs were taken of three portions to represent a small, medium and a large portion. The drink photographs were taken of the bottle/can next to a pint glass which contained the amount from the bottle/ can. The portion sizes of the drinks differed according to the type of drink. While the manufacturers recommended portions for SSBs are 250ml, this portion size is not commonly found in supermarkets. Thus, the SSB portion sizes reflect the typical portion sizes which are available for purchase. For example, a small can of a sugar-sweetened carbonated beverage was 150ml, a medium can was 330ml and a large was 500ml, in comparison to a small serving of full sugar squash, which was 250ml, a medium serving was 288ml, and a large serving was 500ml.

Calculating desired portion size

To identify participants' 'desired' portion size, participants were presented with photographs on the online survey of 24 HED and LED foods, and seven SSBs and non-SSBs. For the

HED snacks participants were presented with four portion sizes (half a portion, one portion, one and a half portions and two portions) and for the SSBs participants were presented with three portion sizes (small, medium and large). The HED foods were presented first, followed by the LED foods, and then the drinks. The order which the food and drinks were presented in was randomised using randomizer.org to ensure that the portion sizes of the foods were evenly distributed, and the same food did not appear twice in a row with a different portion size. Desired portion size was calculated by identifying which portion size participants selected as being 'just right' for each food and drink item. For example, if half a portion was selected as being 'just right' then the desired portion size for that participant was 0.5, whereas if one portion was selected as being 'just right' then the desired portion size was 1, and so on. If participants rated more than one portion size as 'just right' an average of the portions resulted in the 'just right' portion. If all the portions were selected as 'too little' or 'slightly less than I would eat' then the largest portion size (2) was selected, and if all of the portions were selected as 'too much' or 'slightly more than I would eat' then the smallest portion size (0.5) was selected. Following this, a mean desired portion size was calculated for the HED snacks combined and the SSBs combined as two separate variables at the two time points (baseline and intervention end).

Frequency, liking and intentions

Participants were presented with the statements: 'I intend to reduce my portion sizes of high calorie snack food in the near future', 'I intend to reduce my portion sizes of sugar-sweetened beverages in the near future', 'I intend to keep my portions of high calorie snack food the same in the near future', 'I intend to keep my portions of sugar-sweetened beverages the same in the near future'. Participants rated these statements on a 5-point Likert scale from

completely disagree to completely agree. For frequency participants were asked ‘during the past month, how often did you eat this food’ with six response options from ‘less than once per month or never’ (coded as 1) to ‘every day or more than once per day’ (coded as 6). For liking, participants were asked ‘how much do you like this item?’ with five response options (Likert scale) from ‘strongly dislike’ (coded as 1) to ‘strongly like’ (coded as 5).

Unadjusted results

The results have been adjusted for age and BMI (intervention 1)/ zBMI (intervention 2). All means and SDs for all supplementary analyses are reported in supplementary table 1 for intervention 1, and supplementary table 2 for intervention 2.

Intervention 1 results adjusted for age and BMI

Participants' reported portion sizes

The results of the ANOVA showed no significant main effect of food type [F (1, 16) = .15, p =.70, ηp^2 = .01], no significant main effect of time [F (1, 16) = 4.10, p = .06, ηp^2 = .20], and no significant food type*time interaction [F (1, 16) = .17, p = .69, ηp^2 = .01] on participants' desired portion sizes of HED snacks and SSBs between baseline and intervention end. Thus, the results indicate that exposure to the intervention did not influence participants rated desired portions of HED snacks and SSBs. See supplementary Table 1 for desired portion sizes at baseline and intervention end.

Reported perceptions of their peers' portion sizes

There was no significant main effect of food type [F (1, 16) = .95, p = .34, ηp^2 = .06]. There was a significant main effect of time [F (1, 16) = 4.95, p = .04, ηp^2 = .24], whereby, participants perceptions of their peers' portions of HED snacks and SSBs reduced following the intervention compared to baseline. There was no significant food type*time interaction [F (1, 16) = < .001, p = .99, ηp^2 = < .001] on participants' perceptions of their peers' portion sizes between baseline and intervention end. The intervention influenced participants' perceptions of their peers' portion sizes of HED snacks, whereby, participants perceived their

peers to consume smaller portions of HED snacks following the intervention compared to baseline.

Reported frequency of consumption and liking

For frequency of consumption, there was no significant main effect of food type [F (1, 16) = .13, p = .73, ηp^2 = .01], no significant main effect of time [F (1, 16) = 1.10, p = .31, ηp^2 = .06], and no significant food type*time interaction [F (1, 16) = 1.42, p = .25, ηp^2 = .08] on participants' frequency of consumption of HED snacks or SSBs between baseline and intervention end. For liking, there was no significant main effect of food type [F (1, 16) = .98, p = .34, ηp^2 = .06], no significant main effect of time [F (1, 16) = .17, p = .69, ηp^2 = .01], and no significant food type* time interaction [F (1, 16) = .60, p = .45, ηp^2 = .04]. The intervention did not influence participants' reported frequency of consumption or liking of either HED snacks or SSBs. The intervention did not influence participants' frequency of consumption or liking of HED snacks or SSBs.

Intentions

There was no significant main effect of food type [F (1, 16) = 1.44, p = .25, ηp^2 = .08], no significant main effect of time [F (1, 16) = .80, p = .38, ηp^2 = .05], and no significant time*food type interaction [F (1, 16) = .15, p = .71, ηp^2 = .01] on participants' intentions regarding their portion sizes of HED snacks or SSBs. Thus, the intervention did not influence participants' intentions regarding their portion sizes.

Table S1. Participants' mean (SDs) desired portion sizes, perceptions of peers' desired portion sizes, frequency of consumption, liking, and intentions regarding participants' HED snack and SSB intake, adjusted for age and BMI.

	HED snacks (n=19)		SSBs (n=19)	
	Baseline	Intervention end	Baseline	Intervention end
Participants' desired portion size*	1.45 (.29)	1.29 (.27)	.87 (.21)	.79 (.26)
Perceptions of peers' desired portion size*	1.48 (.26)	1.37 (.26)	.86 (.23)	.90 (.25)
Frequency of consumption**	1.58 (.34)	1.52 (.46)	2.18 (.76)	2.00 (.82)
Liking**	4.00 (.39)	3.96 (.31)	3.77 (.65)	3.82 (.44)
Intentions ***	3.13 (.28)	3.08 (.34)	3.16 (.34)	3.13 (.50)

*For desired portion size, a value of 1 refers to the recommended portion size for HED snacks and the typical portion for SSBs. A number greater than 1 indicates the 'desired' portion size is greater than the recommended/ typical portion, and a number smaller than 1 indicates that the 'desired' portion size is smaller than the recommended/typical portion.

** Frequency of consumption was measured on a 6-point Likert style scale from once per month or never to daily. Liking was measured on a 5-point Likert scale from strongly dislike to strongly like.

***Intentions were assessed on a 5-point Likert-style scale from completely disagree to completely agree.

Intervention 2 results adjusted for age and zBMI

Participants' reported portion sizes

There was no significant main effect of condition [F (1, 30) = 2.62, p = .12, ηp^2 = .08], no significant main effect of food type [F (1, 30) = 2.31, p = .14, ηp^2 = .07], and no significant main effect of time [F (1, 30) = 1.46, p = .24, ηp^2 = .05]. There were no significant interactions between condition and food type [F (1, 30) = .18, p = .68, ηp^2 = .01], condition and time [F (1, 30) = .004, p = .95, $\eta p^2 < .001$], and no significant condition*food type*time interaction [F (1, 30) = .62, p = .44, ηp^2 = .02] on participants' desired portion sizes of HED snacks and SSBs between baseline and intervention end. Thus, the intervention did not

influence participants to reduce their desired portion sizes of HED snacks or SSBs relative to the control condition.

Reported perceptions of peers' portion sizes

There was no significant main effect of condition [F (1, 30) = .56, p = .46, ηp^2 = .02], no significant main effect of food type [F (1, 30) = 2.59, p = .12, ηp^2 =.08], and no significant main effect of time [F (1, 30) = .23, p = .63, ηp^2 = .01]. There were no significant interactions between condition and food type [F (1, 30) = 1.23, p = .28, ηp^2 = .04], condition and time [F (1, 30) = .19, p = .67, ηp^2 = .01], food type and time [F (1, 30) = .79, p = .38, ηp^2 = .03], and no significant condition*food type*time interaction [F (1, 30) = 1.34, p = .26, ηp^2 =.04] on participants' perceptions of their peers' portion sizes of HED snacks and SSBs between baseline and intervention end. Thus, the intervention did not significantly influence participants' perceptions of their peers' desired portion sizes of HED snacks or SSBs relative to the control condition.

Reported frequency of consumption and liking

There was no significant main effect of condition [F (1, 30) = .40, p = .53, ηp^2 =.01], no significant main effect of food type [F (1, 30) = .02, p = .89, ηp^2 = .001], and no significant main effect of time [F (1, 30) = 1.16, p = .29, ηp^2 = .04]. There were no significant interactions between condition and food type [F (1, 30) = .07, p = .79, ηp^2 = .001], condition and time [F (1, 30) = .58, p = .45, ηp^2 = .02], food type and time [F (1, 30) = .48, p = .50, ηp^2 = .02], and no significant condition*food type*time interaction [F (1, 30) = .16, p = .69, ηp^2 =.01] on participants' frequency of consumption of HED snacks and SSBs between baseline

and intervention end. For liking, there was no significant main effect of condition [F (1, 30) = .25, p = .62, ηp^2 =.01], no significant main effect of food type [F (1, 30) = .50, p = .49, ηp^2 =.02], and no significant main effect of time [F (1, 30) = 1.20, p = .28, ηp^2 =.04]. There were no significant interactions between condition and food type [F (1, 30) < .001, p = .99, $\eta p^2 <$ .001], condition and time [F (1, 30) = .58, p = .45, ηp^2 = .02], food type and time [F (1, 30) = .14, p = .71, ηp^2 = .01], and no significant food type*time*condition interaction [F (1, 30) = .01, p = .93, ηp^2 = < .001]. Thus, the intervention did not influence participants' reported frequency of consumption or liking.

Intentions

There was no significant main effect of condition [F (1, 29) = .04, p = .84, ηp^2 = .002], no significant main effect of food type [F (1, 29) = 1.00, p = .33, ηp^2 = .03], and no significant main effect of time [F (1, 29) = 1.47, p = .24, ηp^2 = .05]. There were no interactions between condition and food type [F (1, 29) = 3.14, p = .09, ηp^2 = .10], condition and time [F (1, 29) = .05, p = .83, ηp^2 = .002], food type and time [F (1, 29) = .46, p = .50, ηp^2 = .02], and no significant condition*time*food type interaction [F (1, 29) = .32, p = .58, ηp^2 = .01]. Thus, the intervention did not influence adolescents' intentions regarding their portion sizes.

Results of Intervention 1 with the male participant removed

Participants' reported desired portion sizes

There was a significant main effect of time [F (1, 18) = 12.57, p = .002, ηp^2 = .41]. Participants reported smaller desired portion sizes of HED snacks and SSBs at intervention

end than at baseline. There was no significant food type by time interaction [$F (1, 18) = 2.67$, $p = .12$, $\eta p^2 = .13$].

Table S2. Mean (SDs) participants' reports of desired portion sizes, perceptions of peers' desired portion sizes, frequency of consumption, liking, and intentions regarding participants' HED snack and SSB intake adjusted for age and zBMI.

	HED snacks				SSBs			
	Intervention		Control		Intervention		Control	
	Baseline	Intervention end	Baseline	Intervention end	Baseline	Intervention end	Baseline	Intervention end
Participants' desired portion size*	1.22 (.35)	1.22 (.37)	1.38 (.33)	1.40 (.36)	.82 (.22)	.80 (.24)	.95 (.33)	.89 (.33)
Perceptions of peers' desired portion size*	1.36 (.39)	1.35 (.38)	1.44 (.35)	1.52 (.29)	.91 (.28)	.93 (.26)	.93 (.27)	.91 (.30)
Participants' frequency of consumption**	2.07 (.53)	2.07 (.61)	2.05 (.56)	1.91 (.45)	2.28 (.88)	2.24 (.73)	2.15 (.92)	2.06 (.94)
Liking**	4.14 (.49)	4.06 (.56)	4.13 (.56)	3.89 (.91)	3.91 (.81)	3.65 (.97)	3.90 (.92)	3.46 (1.16)
Intentions	3.11 (.37)	3.08 (.60)	3.13 (.58)	3.23 (.32)	3.14 (.45)	3.28 (.60)	3.03 (.23)	3.10 (.43)

*For desired portion size, a value of 1 refers to the recommended portion size of HED snacks and the typical portion size of SSBs. A number greater than 1 indicates the 'desired' portion size is greater than the recommended/ typical portion, and a number smaller than 1 indicates that the 'desired' portion size is smaller than the recommended/typical portion.

** Frequency of consumption was measured on a 6-point Likert style scale from once per month or never to daily. Liking was measured on a 5-point Likert scale from strongly dislike to strongly like.

***Intentions were assessed on a 5-point Likert-style scale from completely disagree to completely agree.
